# Methylsulfonylmethane enhances MSC chondrogenic commitment and promotes pre-osteoblasts formation

**DOI:** 10.1186/s13287-021-02396-5

**Published:** 2021-06-05

**Authors:** Luca Dalle Carbonare, Jessica Bertacco, Giulia Marchetto, Samuele Cheri, Michela Deiana, Arianna Minoia, Natascia Tiso, Monica Mottes, Maria Teresa Valenti

**Affiliations:** 1 Department of Medicine, University of Verona and Azienda Ospedaliera Universitaria Integrata Verona, Verona, Italy; 2 Department of Neurosciences, Biomedicine and Movement Sciences, University of Verona, Strada Le Grazie, 10, 37100 Verona, Italy; 3 Department of Biology, University of Padova, I-35131 Padova, Italy

**Keywords:** Methylsulfonylmethane, Mesenchymal stem cells, Chondrocytes, Osteoblasts

## Abstract

**Background:**

Methylsulfonylmethane (MSM) is a nutraceutical compound which has been indicated to counteract osteoarthritis, a cartilage degenerative disorder. In addition, MSM has also been shown to increase osteoblast differentiation. So far, few studies have investigated MSM role in the differentiation of mesenchymal stem cells (MSCs), and no study has been performed to evaluate its overall effects on both osteogenic and chondrogenic differentiation. These two mutually regulated processes share the same progenitor cells.

**Methods:**

Therefore, with the aim to evaluate the effects of MSM on chondrogenesis and osteogenesis, we analyzed the expression of SOX9, RUNX2, and SP7 transcription factors in vitro (mesenchymal stem cells and chondrocytes cell lines) and in vivo (zebrafish model). Real-time PCR as well Western blotting, immunofluorescence, and specific in vitro and in vivo staining have been performed. Student’s paired t test was used to compare the variation between the groups.

**Results:**

Our data demonstrated that MSM modulates the expression of differentiation-related genes both in vitro and in vivo. The increased SOX9 expression suggests that MSM promotes chondrogenesis in treated samples. In addition, RUNX2 expression was not particularly affected by MSM while SP7 expression increased in all MSM samples/model analyzed. As SP7 is required for the final commitment of progenitors to preosteoblasts, our data suggest a role of MSM in promoting preosteoblast formation. In addition, we observed a reduced expression of the osteoclast-surface receptor RANK in larvae and in scales as well as a reduced pERK/ERK ratio in fin and scale of MSM treated zebrafish.

**Conclusions:**

In conclusion, our study provides new insights into MSM mode of action and suggests that MSM is a useful tool to counteract skeletal degenerative diseases by targeting MSC commitment and differentiation.

## Background

Methylsulfonylmethane (MSM) is a popular dietary supplement, suggested for relieving joint/muscle pain, thanks to its anti-inflammatory properties. MSM is a natural member of the methyl-S-methane compounds, and it can be synthetically produced through the oxidation of DMSO with hydrogen peroxide (H2O2) [1]. In in vitro studies, it has been demonstrated that MSM inhibits the nuclear factor kappa-light-chain-enhancer of activated B cells (NF-κB) [2, 3] as well as p65-S536 phosphorylation [4]. Hence, NF-κB inhibition results in the reduced expression of IL-1, IL-6, tumor, and necrosis factor-α (TNF-α) [5, 6]. In addition, MSM reduces the expression of nitric oxide synthase (iNOS) and cyclooxygenase-2 (COX-2) [7].

MSM has been suggested as a nutraceutical compound to counteract osteoarthritis [8]. Osteoarthritis (OA) is a degenerative disease characterized by pain, affecting cartilage homeostasis by inducing its degradation and impairing matrix neo-synthesis by chondrocytes [9]. Chondrocytes derive from mesenchymal stem cell (MSCs) condensation and differentiation induced by the transcription factor SOX9 [10, 11]. When the balance between degradation and synthesis of cartilage matrix proteins is affected, inflammation and OA disease arise [12]. Cheleschi et al. by performing an in vitro study demonstrated that MSM has beneficial effects on chondrocytes exposed to interleukin (IL)-1β, most likely by modulating the NF-κB pathway [8]. In another in vitro study, MSM was shown to increase osteoblast differentiation through the Jak2/STAT5b pathway [13]. It is well known that MSC osteogenic differentiation is induced by the upregulation of the master gene RUNX2, which in turn transactivates its downstream partner SP7 (Osterix), SP7 then promotes the expression of osteogenic genes such as SPARC (osteonectin), SSP1 (osteopontin), COL1A1 and COL1A2 (collagen type I), and BGLAP (osteocalcin) [14]. Other in vitro studies suggested that MSM increases osteogenic differentiation by inducing BMP2 [15] or transglutaminase-2 (TG2) [16]. MSM influence on the commitment and differentiation of mesenchymal cells appears evident on the basis of these findings. SOX9 and RUNX2 interact in progenitor cells and can affect their respective transactivation in a reciprocal way [17]. Regulation of RUNX2 and SOX9 occurs at the transcriptional and posttranscriptional levels: phosphorylation, acetylation, and ubiquitination can modulate their activity [18–20]. The temporal and spatial distribution and overlapping expression of RUNX2 and SOX9 occurring in embryogenesis show a coordinated interplay between these two genes in bone formation [21–23]. Importantly, it has been demonstrated that SOX9 induces RUNX2 degradation; hence, it plays an important role in controlling mesenchymal stem cells differentiation fate [17].

With the aim to deepen our knowledge about MSM effects on both chondrogenesis and osteogenesis, we have investigated the molecular modulation following MSM supplementation during the differentiation in both in vitro models (mesenchymal stem cells and chondrocytes) and in vivo models (larvae and adult zebrafish).

## Methods

### Metylsulfonylmethane

Metylsulfonylmethane (MSM) (Artrosulfur; Laborest S.r.l, Assago, MI, Italy) was used at the concentration of 20 mM as previously reported [24, 25].

### XTT test

Cell viability was evaluated by using the Cell proliferation kit II (XTT Chemicon) as previously described [26]. Six replicates in three independent experiments were tested.

### Cell cultures

Human mesenchimal stem cells (hMSC, PromoCell, Heidelberg, Germany) were plated at a density of 5 × 10^4^ cells and cultured with mesenchymal stem cell growth medium (PromoCell) or osteogenic differentiation medium or chondrogenic differentiation medium (PromoCell, Heidelberg, Germany) and incubated at 37°C in a humidified atmosphere with 5% CO_2_. Differentiating cells were then used for further analyses. Human chondrocytes (HCH), PromoCell, Heidelberg, Germany) were plated at a density of 5 × 10^4^ cells and cultured with DMEM 10% FBS medium at 37°C with 5% CO_2_. IL1β (1 ng/mL) was added to HCH cells to mimic OA conditions, as previously reported [26]. Three independent experiments were performed for each condition.

### Zebrafish

Zebrafish experiments were performed at the CIRSAL (Interdepartmental Centre of Experimental Research Service) of the University of Verona, Italy. The animal protocol was approved by the Italian Ministry of Health Directorate-General for animal health and veterinary medicinal products (authorization n. 662/2019-PR of 16/09/2019). The embryos were obtained from natural spawning of *nacre* adults (ZFIN database ID: ZDB-ALT-990423-22), according to standard protocols [27], and staged according to Kimmel [28]. Zebrafish embryos were grown at 33°C in water containing 20-mM MSM from 2 days post fertilization (dpf). Zebrafish embryos were treated with MSM up to 1 week (experimental endpoint, 9 dpf) or 2 weeks (experimental endpoint, 16 dpf). At the end of the treatment, zebrafish embryos were euthanized and collected for molecular analyses as described below. Calcein staining was performed as previously reported [29]. Imaging was performed using Leica M205FA fluorescence microscope (Leica Microsystems, Wetzlar, Germany). Stained areas were quantified by using the ImageJ software, as previously reported [29].

Adult zebrafish (15–20 months) were grown in water containing 20 mM of MSM for 14 weeks. At the experimental endpoint, zebrafish were euthanized and collected for staining and molecular procedures, as reported below.

### Total RNA extraction and reverse transcription

Pellets from cell lines or zebrafish samples were collected, stored at −80°C and processed for RNA extraction with a “RNeasy® protect mini kit” (Qiagen, Hilden, Germany) following the manufacturer’s instruction. RNA samples were quantified using the Qubit™ RNA HS assay kit” (Invitrogen, Carlsbad, USA). RNA (1 mg) was reverse transcribed using the first-strand cDNA synthesis kit (GE Healthcare, Little Chalfont), according to the manufacturer’s instruction. miRNAs were extracted by using miRNeasy Mini Kit (Qiagen, Hilden, Germany), according to the manufacturer’s instructions. First-strand complementary DNA (c-DNA) synthesis was performed using the TaqMan™ MicroRNA Reverse Transcription Kit (Applied Biosystems, CA, USA) according to the manufacturer’s protocol. RNA, microRNAs, and cDNA samples were stored at −80°C until use.

### Real-time PCR

To investigate gene expression modulation, we performed real-time PCR analyses as reported previously [30].

Briefly, predesigned, gene-specific primers, and probe sets for each gene (RUNX2, hs00231692_m1; OSTERIX (SP7), hs00541729_m1; COLLAGEN, TYPE I, ALPHA 2 (COL1A2), hs01028956_m1; OSTEONECTIN (SPARC), hs00234160_m1; OSTEOPONTIN (SPP1), hs00167093_m1, SOX9, hs01107818_m1; COMP, hs004359-m1; COL2A1, hs00264051_m1; B2M, hs999999_m1 (housekeeping); GAPDH, 0802021 (housekeeping); sox9a, Dr03112282_m1; col2a1a, Dr03099270_m1; miR-146b-5p, 474220; RNU44, 001094) were obtained from assay-on-demand gene expression products (Thermo Fisher Corporation, Waltham, MA, USA). In addition, the following custom primer sets (Invitrogen, Carlsbad, CA, USA) were also used: runx2a (fw GACGGTGGTGACGGTAATGG, rv TGCGGTGGGTTCGTGAATA), runx2b (fw CGGCTCCTACCAGTTCTCCA, rv CCATCTCCCTCCACTCCTCC), rank (fw GCACGGTTATTGTTGTTA, rv TATTCAGAGGTGGTGTTAT), and as housekeeping gene actb1 (fw CCCAAAGCCAACAGAGAGAA, rv ACCAGAAGCGTACAGAGAGA).

Ct values for each reaction were determined using TaqMan SDS analysis software (Applied Biosystems; Foster City, California, USA) as reported previously. To calculate relative gene expression levels between different samples, we performed the analyses by using the 2−ΔΔCT method as previously reported [30].

### Immunofluorescence

Immunofluorescence analyses were performed as previously reported [30]. Briefly, cells were fixed and processed according to the manufacturer’s protocols. Primary antibodies for RUNX2 and osteocalcin (sc74495, Santa Cruz, Dallas, Texas, USA) were diluted according to the manufacturer’s instruction in antibody dilution buffer and incubated overnight at 4°C. Slides were then incubated with the secondary antibodies goat mouse fluorescein conjugated (cat. Ap124f, Millipore, Burlington, Massachusetts, USA). Nuclear staining was performed by ProLong™ Gold Antifade Mountant with DAPI. The staining was analyzed using a Leica (Wetzlar, Germany) TCS SP5 AOBS microscope. To express data in a semiquantitative way, six different fields were analyzed for each sample, in three independent experiments with about 80–100 total cells.

### Western blotting

Protein levels were separated by SDS Page and investigated by Western blot analyses as previously reported [31]. Briefly, proteins were extracted by RIPA buffer (Thermo Fisher Scientific, Waltham, MA, USA) and protein concentration was established by BCA assay (Thermo Fisher Scientific, Waltham, MA, USA). After protein separation by sodium dodecyl sulfate-polyacrylamide gel electrophoresis (SDS PAGE), proteins were transferred onto polyvinylidene difluoride (PVDF) membranes (Thermo Fisher Scientific, Waltham, MA, USA). PVDF membranes were then probed with the primary (Aggrecan (BC-3), Thermo Fisher Scientific; β-actin (BA3R), Thermo Scientific; ERK (13F5), Cell Signaling; p_ERK (D13.14.4E), Cells Signaling) and secondary antibodies (Anti-mouse (Cell Signaling, 7076); Anti-rabbit (Cell Signaling 7074)). Signals were detected using a chemiluminescence reagent (ECL, Millipore, Burlington, MA, USA) according to the manufacturer’s instructions. Images were acquired by a LAS4000 Digital Image Scanning System (GE Healthcare, Little Chalfont, UK). The Densitometric analysis was performed by ImageQuant software (GE Healthcare, Little Chalfont, UK). Proteins optical density was normalized to β-actin.

### Alizarin Red Staining in in vitro experiments

To evaluate calcium deposition in differentiating osteogenic cells, we performed Alizarin red staining as previously described [30]. Briefly, after 21 days of culture in osteogenic medium, cells were fixed with 70% ethanol and washed with water. Then, cells were stained with 40 mM Alizarin red S for 5 min at pH 4.1 and rinsed for 15 min with 1x phosphate-buffered saline. The stained area was quantified by using ImageJ software (NIH, Bethesda, MD, USA) as previously reported [30]. Six independent experiments were performed.

### Zebrafish staning

Calcein staining in zebrafish larvae was performed as previously reported [29]. Imaging was performed using Leica M205FA fluorescence microscope (Leica Microsystems, Wetzlar, Germany). The stained area was quantified by using the ImageJ software, as previously reported [29].

Bone and cartilage staining was performed on adult (>1 year old) zebrafish according to Sakata-Haga et al. [32], strictly following their “RAP System” protocol. Briefly, euthanized fish were immersed into the FIXATIVE (5% formalin, 5% Triton X-100, 1% potassium hydroxide (KOH) and gently rocked for 12 h at room temperature (RT)). Then, we proceeded either to the cartilage staining step for cartilage-only/double staining or directly to the bone staining step for bone-only stain. For cartilage staining, specimens were immersed into C-STAINING SOLUTION (70% ethanol, 20% acetate, 0.015–0.02% alcian blue) overnight at 20°C, then washed in C-STAINING MEDIUM (containing 1% Triton X-100) overnight at 20°C and washed with 50–70% ethanol. For cartilage-only staining, the protocol proceeded directly to clearing and stock steps. For cartilage and bone double staining, the protocol proceeded to the bone staining step. Briefly, for bone staining, specimens were immersed into B-STAINING MEDIUM (20% ethylene glycol and 1% KOH) and then in “B-STAINING SOLUTION (0.05% alizarin red S, 20% ethylene glycol, 1% KOH) overnight at 20°C. The specimens were then washed with clearing solution (20% Tween 20, 1% KOH) while gentle rocking for 12h, and the stocking was performed in glycerol 100%. All needed solutions were prepared as indicated in Sakata-Haga et al. [32]. The stained area was quantified by using the Image J software, as previously reported [29].

### Statistical analysis

Results were expressed as mean ± S.E. Student’s paired t test was used in order to compare the variation in a variable between two groups. Differences were considered statistically significant with p < 0.05. The statistical analyses were performed using GraphPad Prism software program (version 8.3; GraphPad Software).

## Results

### MSM improves chondrogenesis in vitro and in vivo

**Fig. 1:**
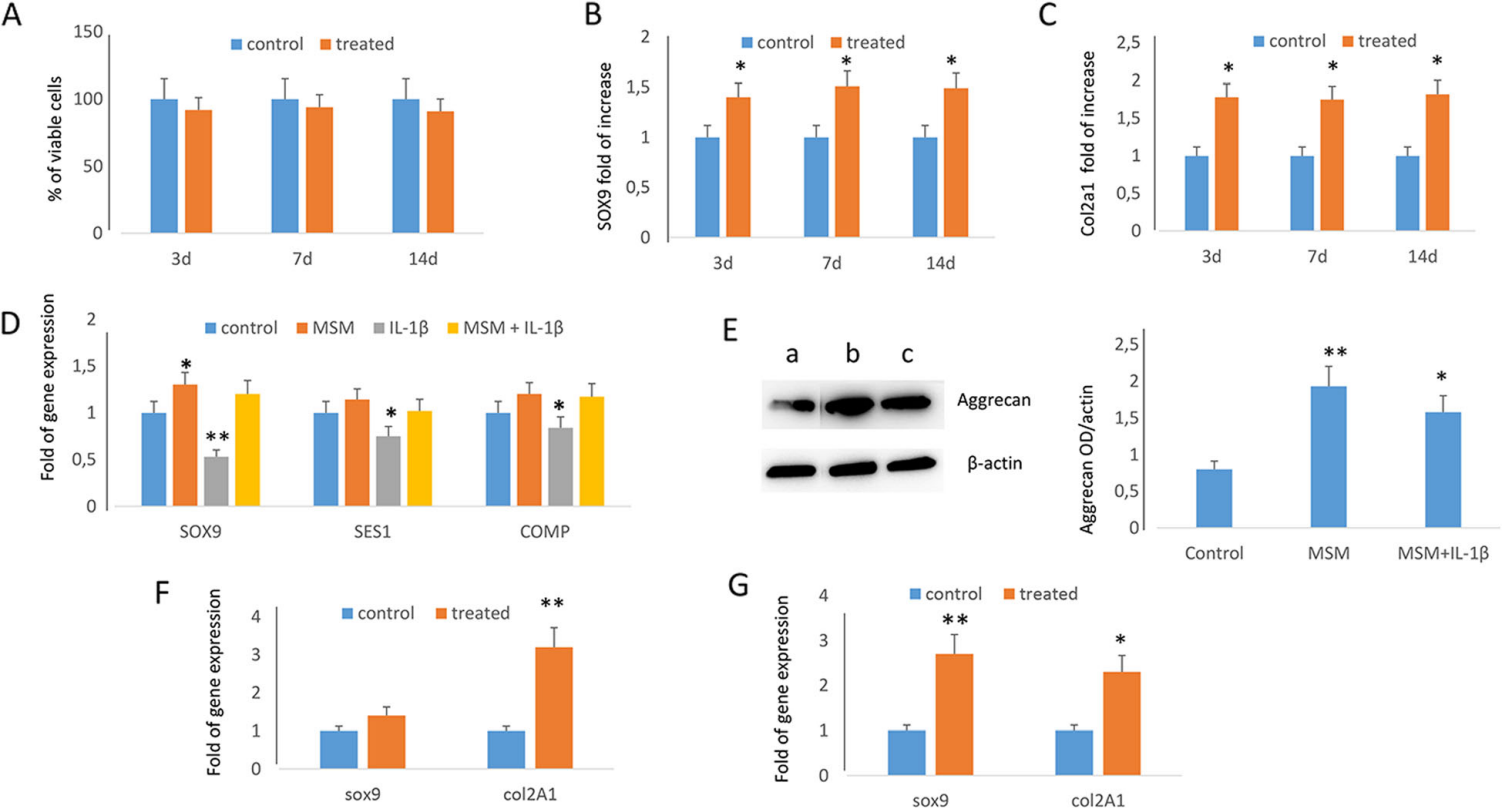
MSM improves chondrogenesis. MSM addition did not affect the cells number (**A**); it upregulated the chondrogenic transcription factor SOX9 (**B**) and COL2A1 expression (**C**) during chondrogenic differentiation. MSM enhanced SOX9 expression in chondrocytes and restored the negative effects of IL-1β (**D**). MSM supplement increased the production of aggrecan by chondrocytes, even in the presence of IL-1β (**E**) (a: control; c: MSM treated; c: IL-1β + MSM treated). MSM upregulated the expression of chondrogenic genes in zebrafish larvae after 7 (9 dpf) (**F**) and 14 (16 dpf) (**G**) days of treatment. *p<0.05; **p<0.01

To evaluate the effects of MSM in chondrogenesis, we cultured mesenchymal stem cells with and without MSM during chondrogenic differentiation. MSM addition to chondrogenic medium did not affect cells number during the differentiation period (Fig. 1A). Instead, we observed the upregulation of the chondrogenic transcription factor SOX9 (Fig. 1B) and of COL2A1 (collagen type II) expression (Fig. 1C) after 3, 7, and 14 days of chondrogenic differentiation.

**Fig. 2:**
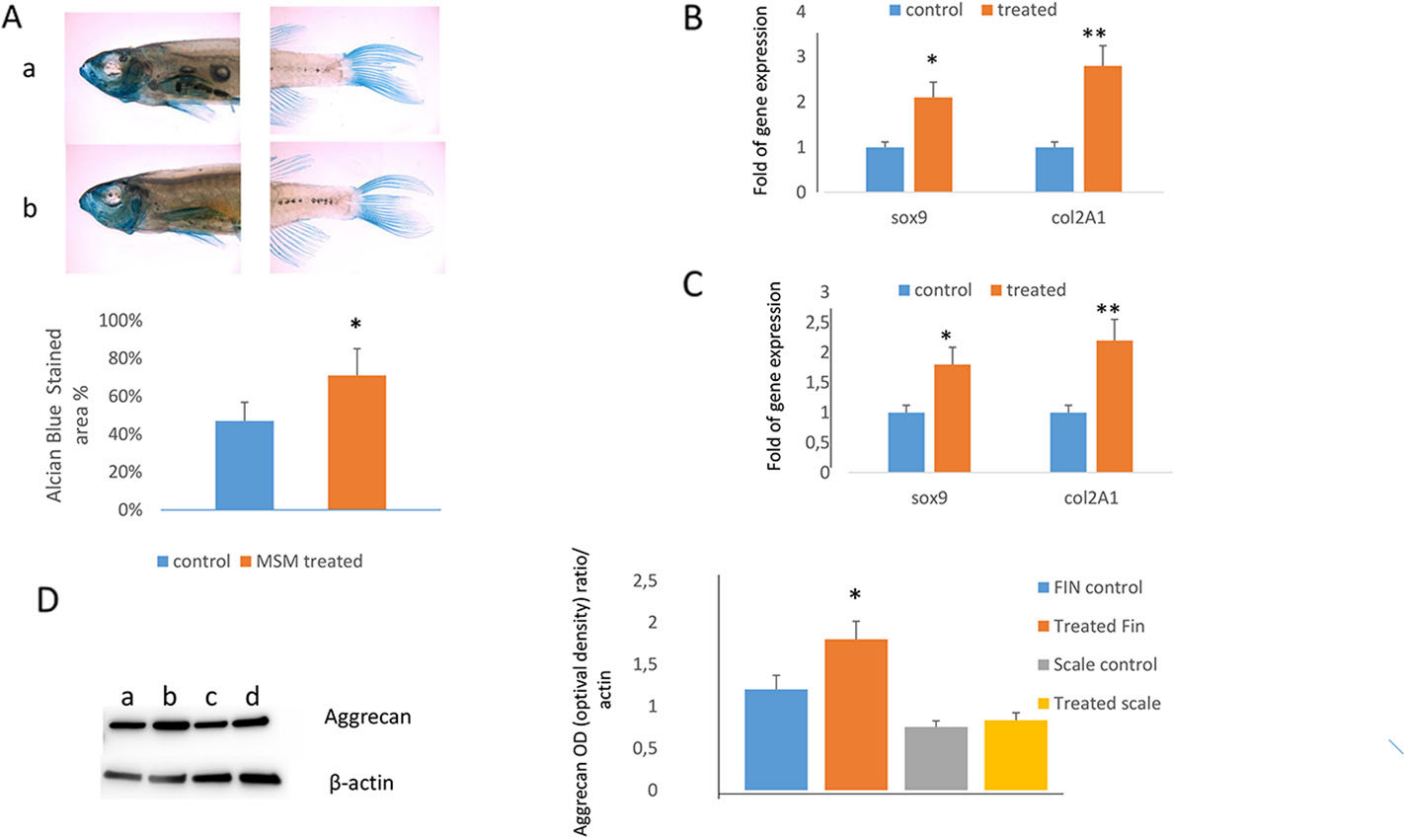
MSM increases chondrogenic gene expression in middle-aged zebrafish. Alcian blue staining was enhanced in MSM-treated zebrafish (**A**) and the expression of chondrogenic genes was increased in fin (**B**) and in scale (**C**) after 14 days of treatment. Aggrecan protein levels increased in MSM-treated zebrafish fin (**D**) while no difference was observed in scale (**D**) (a: control fin; b: treated fin; c: control; d: treated scale). *p<0.05; **p<0.01

Increased SOX9 expression was also observed in chondrocytes cultured for 7 days in the presence of MSM (Fig. 1D). In addition, MSM supplementation restored the negative effects of IL-1β on chondrocytes. In fact, MSM counteracted SOX9 downregulation as well as Sestrin 1 and COMP reduced expression induced by IL-1β (Fig. 1D). Accordingly, the production of aggrecan, a cartilage specific proteoglycan protein, increased in chondrocytes in the presence of MSM, and this effect was appreciable also in chondrocytes treated with IL-1β (Fig. 1E). The upregulation of chondrogenic genes was observed in MSM-treated zebrafish larvae after 7 (9 dpf) (Fig. 1F) and 14 (16 dpf) days (Fig. 1G) of treatment. We thereafter analyzed the effects of MSM treatment on middle-aged zebrafish (15–20 months corresponding to 36–48 years of human age) and observed increased chondrocyte-specific Alcian blue staining in treated zebrafish compared to control (Fig. 2A).

The upregulation of chondrogenic genes was observed after 14 days in fin (Fig. 2B) and in scale (Fig. 2C) of MSM-treated zebrafish. Therefore, as aggrecan plays an important role in cartilage maintenance, we investigated MSM effects on aggrecan production. In particular, we analyzed the protein levels in fin and we observed an increased production in MSM-treated zebrafish compared to controls (Fig. 2D). On the contrary, we did not observe a significantly increased production in zebrafish scale upon MSM treatment (Fig. 2D).

### MSM promotes osteoblast maturation

**Fig. 3:**
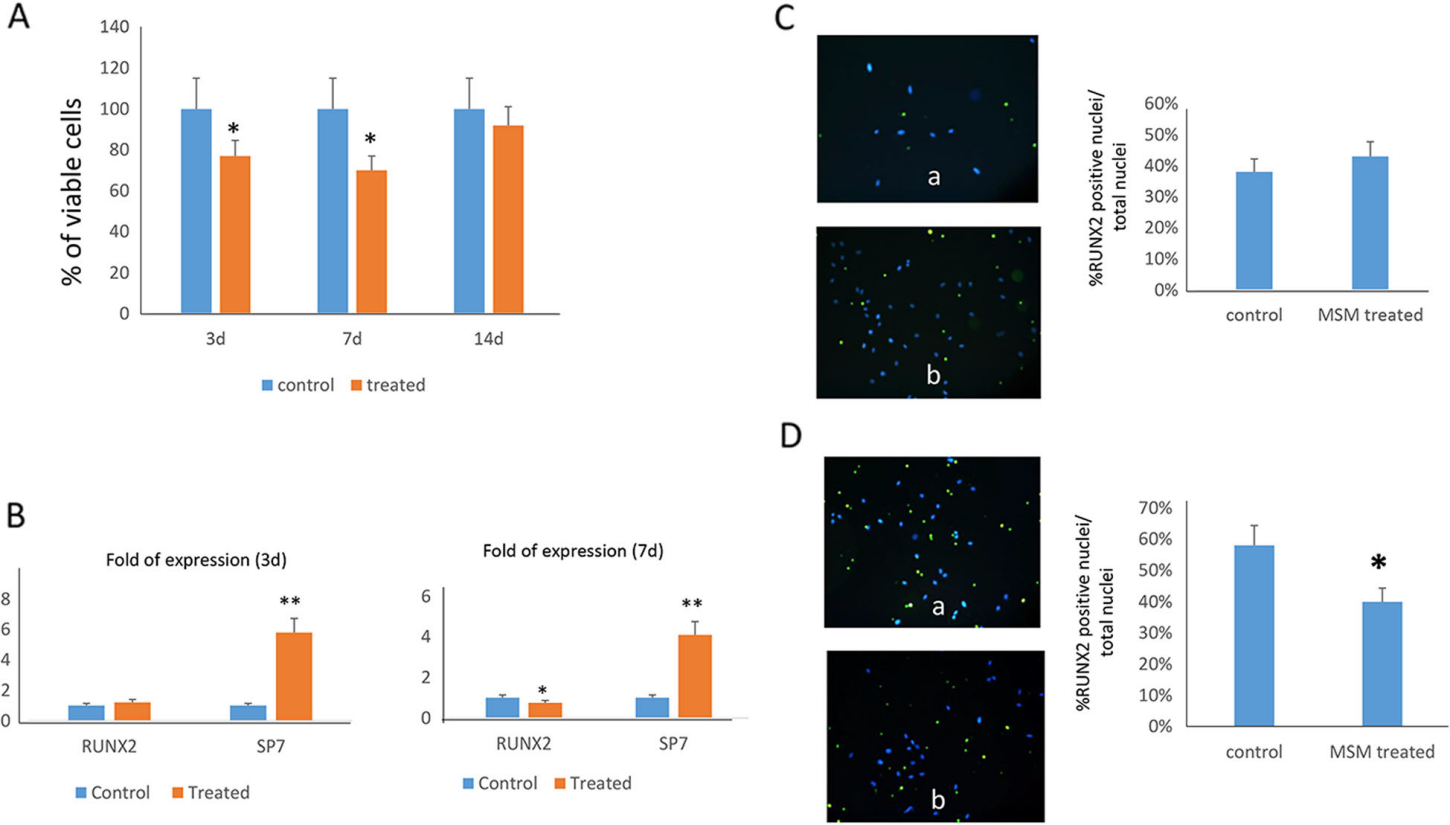
Effects of MSM in the early phase of osteogenic differentiation. After 3 and 7 days of osteogenic differentiation a reduced number of differentiating cells was observed (**A**). In MSM-treated cells SP7 gene expression was upregulated after 3 and 7 days of differentiation whereas RUNX2 was downregulated after 7 days of differentiation (**B**). RUNX2 protein levels were similar after 3 days of osteogenic differentiation (**C**) and significantly lower after 7 days of differentiation (**D**) in nuclei of MSM treated cells. *p<0.05; **p<0.01

**Fig. 4:**
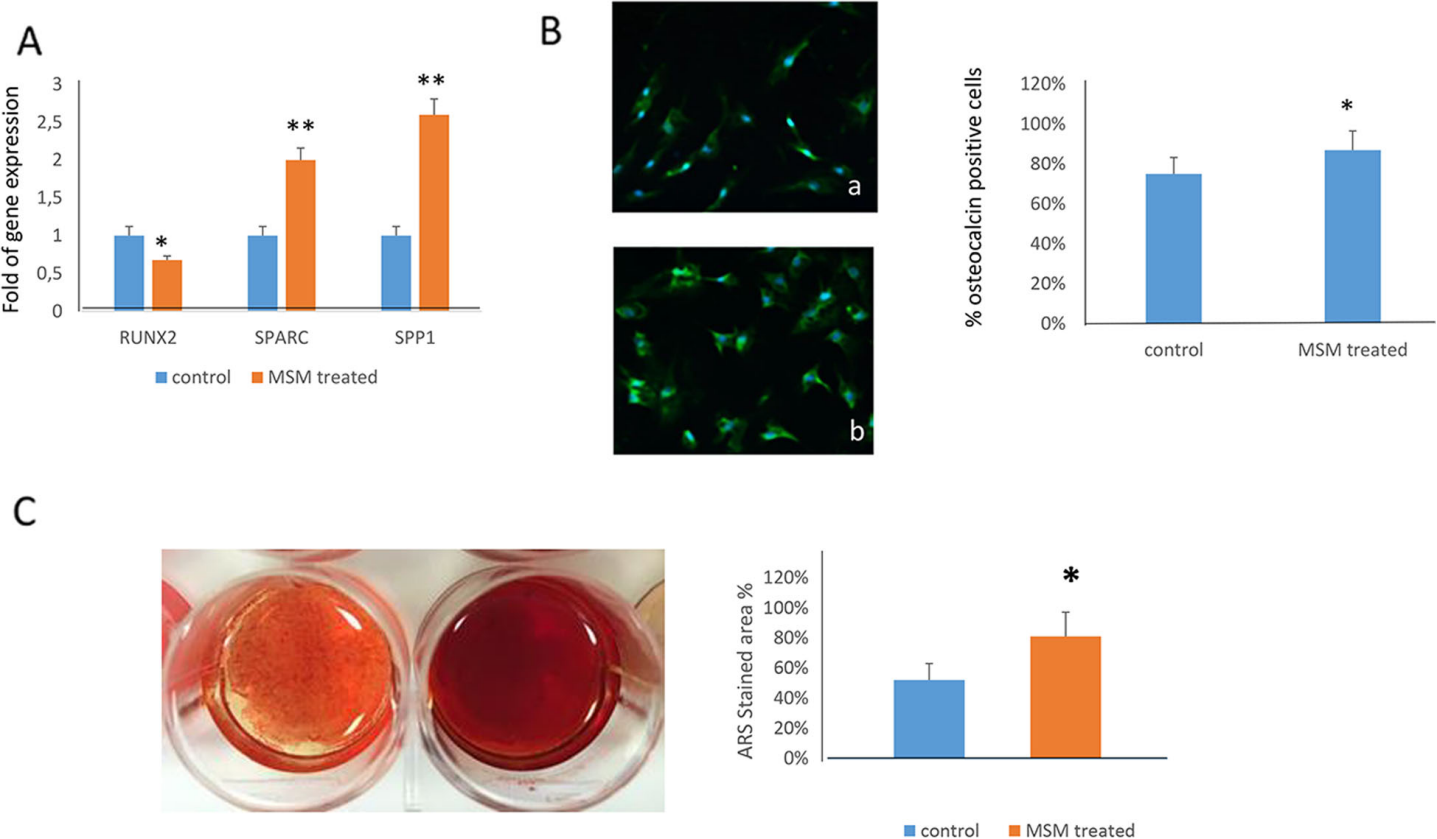
Effects of MSM in the osteogenic maturation. RUNX2 gene expression was reduced while the expression of SPARC and SPP1 (**A**), as well the number of osteocalcin positive cells (**B**) increased in MSM-treated cells after 14 days of osteogenic differentiation. After 21 days of differentiation calcium deposition, evaluated by alizarin red staining (ARS), increased in cells treated with MSM (**C**). *p<0.05; **p<0.01

Considering that chondrogenesis and osteogenesis are intertwining processes, we evaluated the effects of MSM in MSCs during osteogenic differentiation. As shown in Fig. 3A, after 3 and 7 days of osteogenic differentiation, respectively, we observed a reduced number of differentiating cells. Therefore, we analyzed the expression of osteogenic transcription factors RUNX2 and its downstream partner SP7. As shown in Fig. 3B, we observed the upregulation of SP7 in MSM treated cells, whereas RUNX2 expression levels were unchanged after 3 days of osteogenic differentiation. Subsequently, after 7 days of differentiation RUNX2 gene expression was reduced in MSM treated cells, while SP7 higher expression persisted. RUNX2 protein levels were consistently unchanged after 3 days of osteogenic differentiation (Fig. 3C) and significantly reduced after 7 days (Fig. 3D) in MSM-treated cell nuclei. MSM effects on osteogenic differentiation persisted also after 14 and 21 days. As shown in Fig. 4, the expression of RUNX2 was reduced and the expression of osteoblast maturation related genes, e.g., SPARC and SPP1, as well as the number of osteocalcin-positive cells increased in MSM-treated cells after 14 days of osteogenic differentiation (Fig. 4A, B). Therefore, after 21 days of differentiation, we observed an increased calcium deposition, evaluated by alizarin red staining, in cells treated with MSM (Fig. 4C).

### MSM enhances runx2b gene expression in zebrafish larvae

**Fig. 5:**
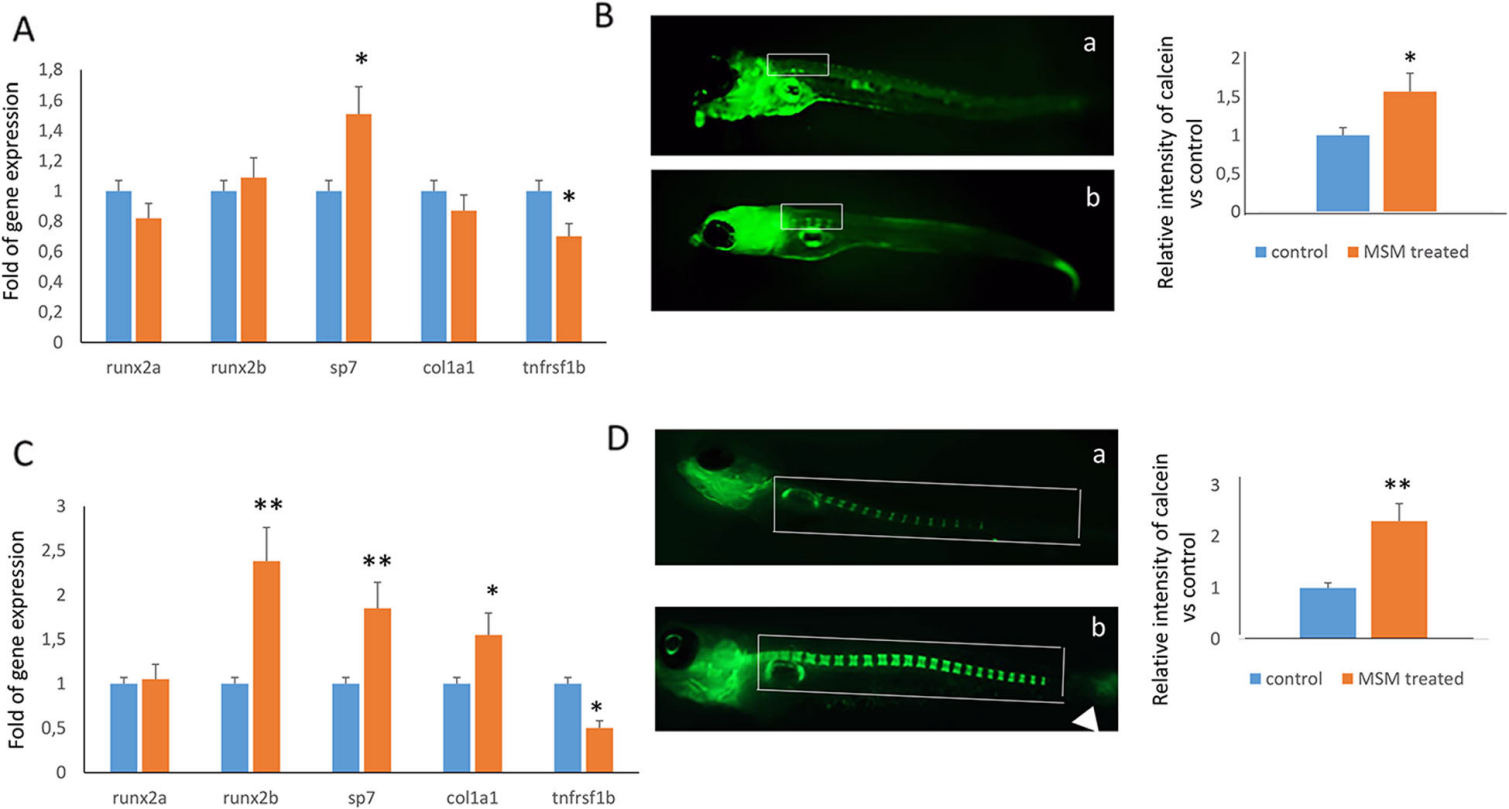
MSM modulates gene expression in zebrafish larvae. Increased expression of *sp7* gene and reduced *tnfrsf1b* gene expression in larvae at 9 dpf (**A**). The fluorescence density produced by calcein staining was higher in larvae after 7 days of MSM treatment compared to untreated larvae (**B**) (a: control; b: MSM treated). Increased *runx2b*, *sp7*, *col1a1*, and reduced *tnfrsf1b* expression observed after 14 days of MSM treatment compared to control (**C**). Bone mineralization evaluated by calcein staining was higher in MSM-treated larvae compared to untreated larvae after 14 days of treatment as well (5D) (a: control; b: MSM treated). *p<0.05; **p<0.01

In order to confirm the effects of MSM on osteogenic maturation, we analyzed the osteogenic markers in zebrafish larvae after 7 and 14 days of treatment in the presence of 20-mM MSM. In particular, zebrafish larvae at 2 days after fertilization (dpf) were treated with or without MSM for 7 or 14 days. After 7 days of treatment, we observed increased expression of *runx2b* and *sp7* genes and reduced tnfrsf1b (homolog of human *RANK*) gene expression (Fig. 5A). To evaluate the direct effects of MSM on bone formation, we quantified bone mineralization by calcein staining. Fluorescence density was observed under microscopic inspection and analyzed by digital methods. Figure 5B shows higher bone mineral density in MSM-treated larvae compared to untreated larvae. Increased expression of *runx2b* and *sp7* and reduced expression of tnfrsf1b were confirmed after 14 days of MSM treatment (Fig. 5C). In addition, we also observed the increased expression of *col1a1* gene. Bone mineralization evaluated by calcein staining was higher in MSM-treated zebrafish compared to untreated zebrafish after 14 days of treatment too (Fig. 5D).

**Fig. 6:**
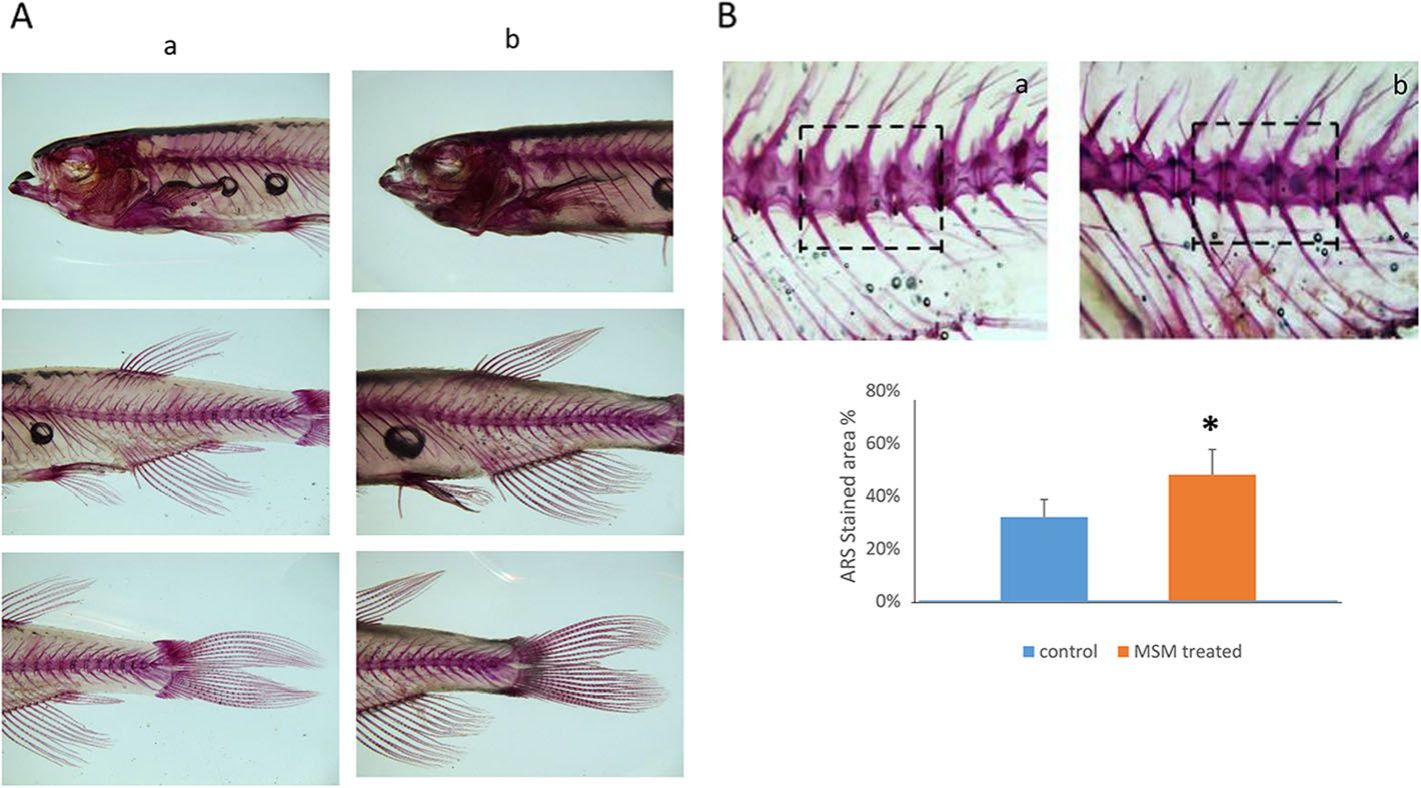
MSM increases mineral deposition in middle-aged zebrafish. Alizarin red staining (ARS) in adult zebrafish untreated (a) and treated (b) with MSM for 14 days. **A** Magnification 4×. Increased percentage of the positive ARS staining area in MSM-treated zebrafish (b) compared to untreated zebrafish (a). **C** Magnification 20×. *p<0.05;

**Fig. 7:**
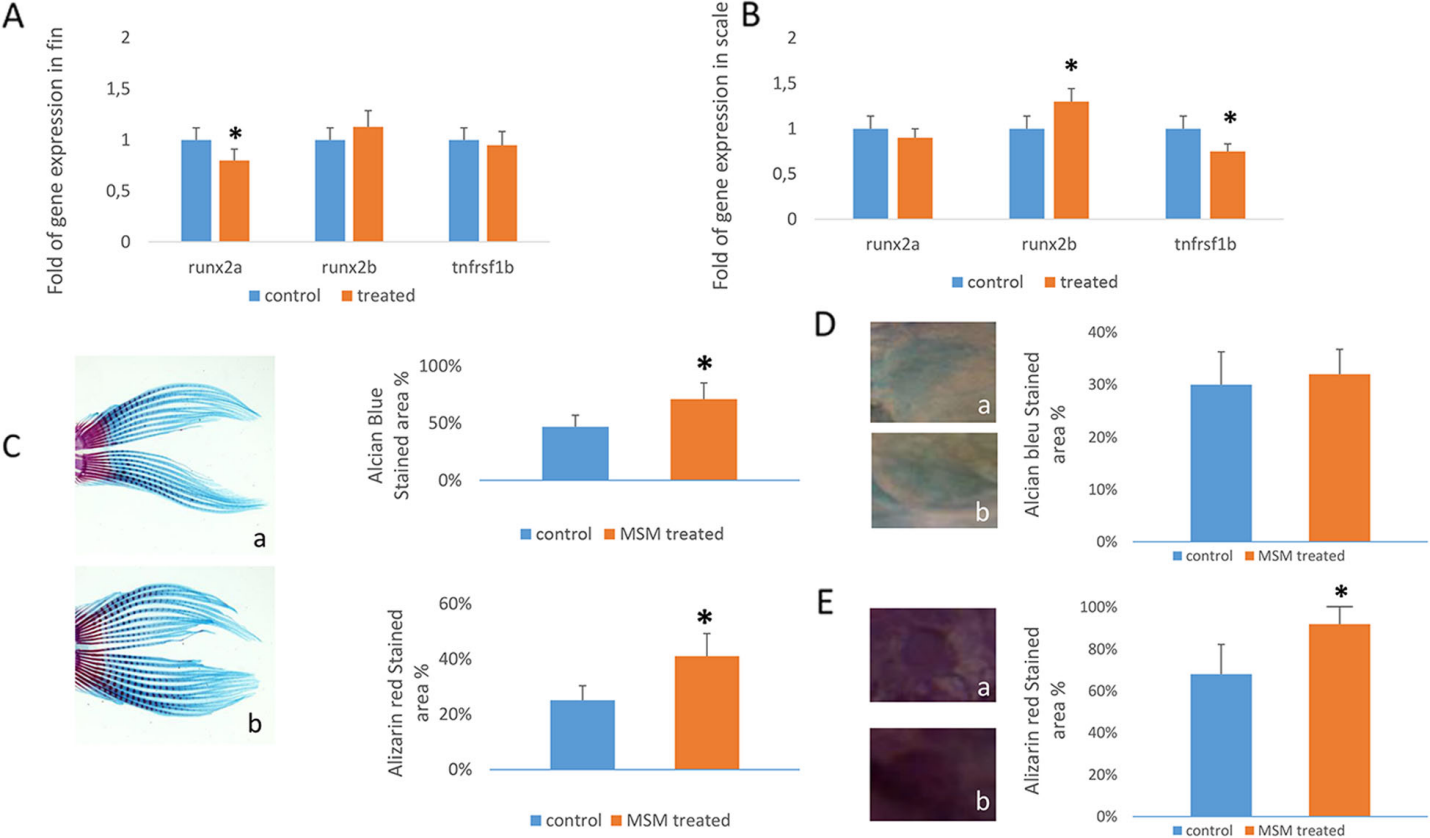
MSM modulates gene expression in middle-aged zebrafish. *Runx2a* gene expression was downregulated in fin of MSM treated adult zebrafish (**A**); *runx2b* expression increased while *tnfrsf1b* expression decreased in scales of MSM-treated zebrafish compared to controls (**B**). Stronger Alcian blue as well as alizarin red staining areas were observed in fin of MSM-treated zebrafish (b) compared to control (a) (**C**); Magnification 4×. The Alcian blue staining was weak in scales of both control (a) and treated (b) zebrafish (**D**) whereas a stronger alizarin red staining area was observed in scales of MSM-treated zebrafish (**E**); Magnification 40×. *p<0.05

Increased bone mineralization, evaluated by alizarin red staining (ARS), was observed also in adult zebrafish treated with MSM for 14 days (Fig. 6A). As shown in Fig. 6B, a more intense ARS and an increased percentage of positive ARS staining area was observed in MSM-treated zebrafish compared to untreated zebrafish (Fig. 6C). In adult zebrafish, we also analyzed the osteogenic gene expression and we observed different gene modulation in fin and scale samples, respectively. As shown in Fig. 7A, r*unx2a* was downregulated in fin of MSM-treated adult zebrafish. On the contrary, we observed increased *runx2b* and reduced tnfrsf1b gene expression in scales of MSM treated zebrafish (Fig. 7B).

To understand the different osteogenic genes modulation, we analyzed Alcian blue and alizarin red staining areas for the evaluation of cartilage and calcium deposition, respectively. As observed in Fig. 7C, a more pronounced Alcian blue area was present in fin of MSM-treated zebrafish compared to control. This finding is consistent with the increased aggrecan levels observed previously in fin of MSM-treated zebrafish. However, we also observed an increased alizarin red staining area in them. On the contrary, Alcian blue staining was weak in scale of both control and treated zebrafish (Fig. 7D) while MSM increased calcium deposition, evaluated by alizarin red staining (Fig. 7E).

**Fig. 8:**
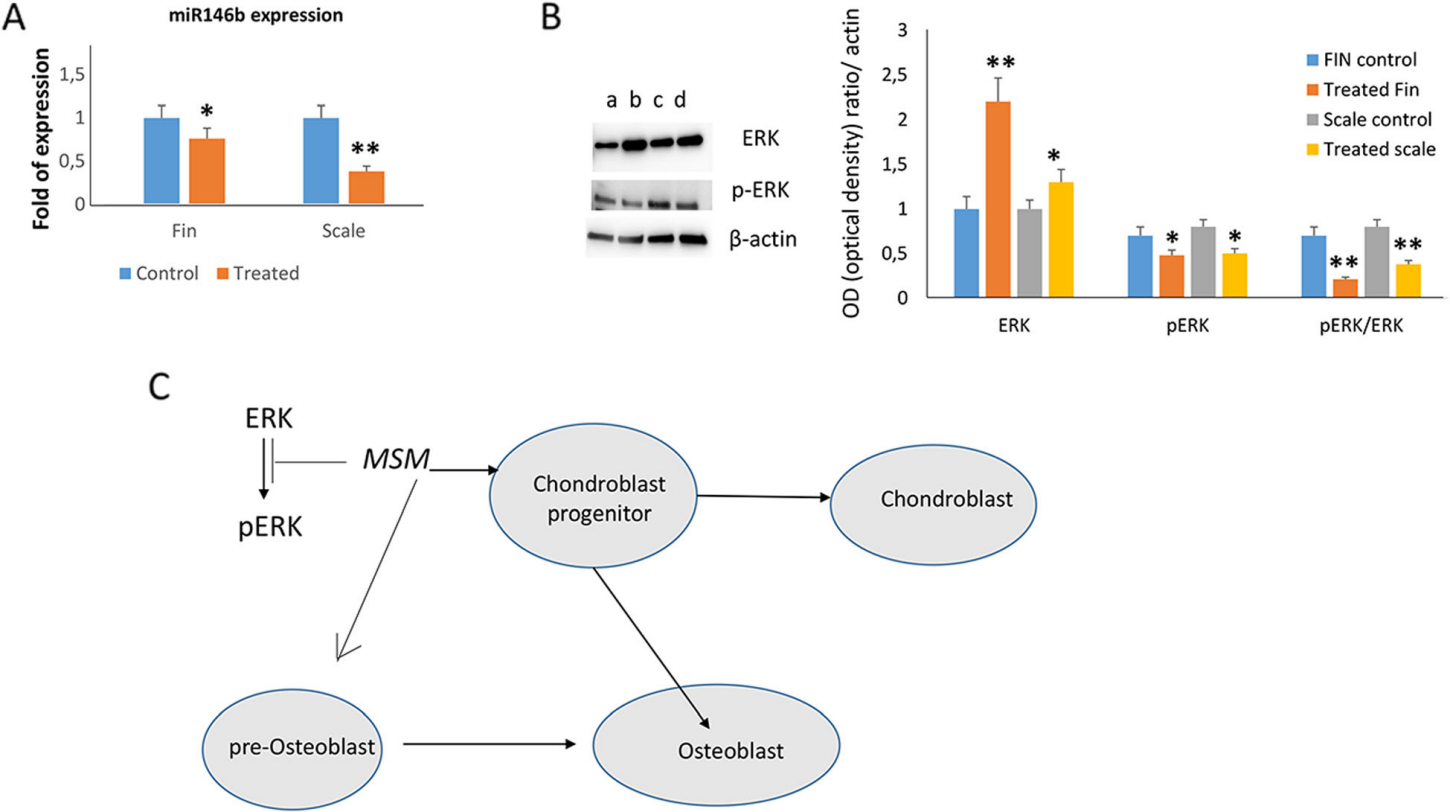
MSM affects miR146b expression and ERK signaling. MiR 146b expression was reduced in both fin and scale treated with MSM (**A**). Increased ERK levels and reduced pERK levels and pERK/ERK ratio were observed in both fin and scale of MSM-treated zebrafish; a: control fin; b: treated fin; c: control scale; d: treated scale (**B**). A schematic graphic showing as MSM promotes chondroblast progenitors and pre-osteoblast maturation by reducing ERK phosphorylation (**C**).

In order to evaluate a possible MSM-epigenetic effect, we analyzed the modulation of miR146b that in humans has been observed to be involved in mesenchymal stem cells differentiation. As shown in Fig. 8A, miR 146b expression was reduced in both fin and scale treated with MSM. Furthermore, as extracellular signal-regulated kinase (ERK) is involved in both osteogenic and chondrogenic commitment, we analyzed the ERK activation pathway in fin and scale of MSM-treated zebrafish. As shown in Fig. 8B, we observed increased levels of ERK and a reduction of pERK as well as of pERK/ERK ratio in both fin and scale of MSM-treated zebrafish.

## Discussion

In the last decades, human lifespans have been increasing; consequently, aging-related diseases such as osteoporosis and osteoarthritis have been increasing in turn. Therefore, the characterization and testing of compounds capable of reducing the degenerative effects of aging-related disorders is an important challenge. In this context, some studies have indicated that MSM may be useful to counteract inflammation-related diseases typical of the aging process. The interest in evaluating MSM effects on progenitor cells should be clear, since mesenchymal stem cells are the precursors of chondrocytes and osteoblasts, the cells which build the cartilage and bone, respectively, tissues which are mainly damaged by the aging process. Nevertheless, a few studies are available, which focused on evaluating the effects of MSM on osteogenic differentiation [24, 25, 33, 34] or on preventing cartilage degradation [35–38]. These studies showed that MSM is capable of enhancing osteogenic differentiation as well as of preventing chondrocytes degradation. However, no study has ever been performed to evaluate MSM overall effects on progenitor cells and to understand how MSM may promote both osteogenic and chondrogenic differentiation. This is a particularly relevant aspect, since osteogenesis and chondrogenesis originate from the same progenitor cells and are highly regulated processes [39].

In our approach to understand the effects of MSM on both chondrogenic and osteogenic processes, we observed an increased chondrogenic differentiation in MSM-treated MSCs. In fact, we observed the upregulation of the chondrogenic transcription factor SOX9, as well as the increased expression of COL2A1, the cartilage ECM marker protein, upon differentiation. The upregulation of SOX9 was also observed in chondrocytes treated with MSM. In addition, we observed the increased expression of SOX9, Sestrin 1, and COMP, the cartilage oligomeric matrix protein [40], also in chondrocytes treated with IL-1β, thus confirming MSM protective role against chondrocyte degradation. It has been demonstrated that Sestrins (sesn) expression is suppressed in chondrocytes of osteoarthritic cartilage. Sestrins increase chondrocyte survival under stress conditions: sesn1 and sesn2 counteract oxidative stress damage by activating the Nrf2 (nuclear factor erythroid 2-related factor 2)-Keap1 (Kelch-like ECH-associated protein 1) pathway [41]. Accordingly, in our study, MSM increased the expression levels of ACAN (aggrecan) in chondrocytes even in the presence of IL-β1, compared to controls. Such finding is noteworthy as aggrecan depletion predisposes to cartilage erosion [42]. In order to investigate whether MSM induces ACAN expression also in vivo, we evaluated the effects of MSM treatment in adult middle-aged zebrafish. Interestingly, we observed increased aggrecan levels in fin of treated zebrafish, but aggrecan expression was not likewise affected in scale. Therefore, our results confirmed that MSM induces chondrogenic differentiation in MSCs and that it counteracts IL1β-negative effects on chondrocytes. In addition, increased aggrecan levels in MSM-treated adult zebrafish fins suggest that MSM enhances cartilage matrix synthesis. The more pronounced aggrecan synthesis in fin is likely due to a higher number of progenitor cells in fin compared to scale. It is well known that fin, which is composed by multiple bony rays, contains nerves, blood vessels, and mesenchymal cells [43] whereas scale contains bone-forming cells (osteoblasts) and bone-resorbing cells (osteoclasts) [44].

MSM treatment affected osteogenic differentiation both in vitro and in vivo. We observed increased expression of RUNX2-induced genes, whereas RUNX2 own expression was reduced in MSM-treated cells. The number of MSCs was reduced as well. SP7 expression increased during osteogenic route in MSM-treated samples. Since SP7 is mandatory for the final commitment of progenitors to preosteoblasts [45], our data suggest a role of MSM in promoting pre-osteoblast formation. The increased percentage of osteocalcin-positive cells, as well as calcium deposition increase evaluated by AR staining in MSM-treated cells, suggest MSM role in promoting osteoblasts maturation rather than osteogenic commitment. The induction of osteogenic maturation was observed also in the zebrafish model where increased calcein staining was observed after 7 and 14 days of treatment. Interestingly, we observed the increased gene expression of *runx2b* whereas *runx2a* was not affected after 14 days of MSM supplementation. Flores et al. demonstrated that *runx2b* is expressed in the vertebral column and in the fin in a juvenile fish and that *runx2a* is expressed in mesenchymal cells during caudal fin regeneration [46]. Therefore, the higher modulation of *runx2b* versus r*unx2a* observed in MSM-treated zebrafish suggests the effects of MSM on mature osteoblasts rather than on MSC commitment consistently with the RUNX2 downmodulation observed in in vitro experiments with mesenchymal stem cells.

Runx2b regulates SP7/osterix and osteocalcin expression indicating its central role in skeletal development [47, 48]. The increased osteogenic maturation was observed in MSM-treated adult zebrafish; a different gene expression pattern was observed in fin or scale of MSM-treated zebrafish. The increased *runx2b* and reduced *tnfrsf1b* expression observed in scale but not in fin reflect (as for aggrecan seen above) the different cellular composition of the two structures, i.e., the prevalence of osteoblasts and osteoclasts in scale. However, further more prolonged treatment, demonstrated increased runx2b expression even in fin. The reduced pERK and pERK/ERK ratio together with the increased ERK levels suggest the inhibition of Mitogen-activated protein kinases (MAPKs), the proteins involved in ERK phosphorylation [49]. As the reactive oxygen species (ROS) activate MAPK pathways [50], we speculate that the reduced ERK phosphorylation might be a consequence of ROS reduction due to MSM treatment. In fact, it is well known that MSM reduces mitochondrial ROS generation [51]. Interestingly, we observed a reduced miR146b expression in MSM-treated fin and scale. The miR-146 family is highly conserved between fish and humans, and it shares the same seed sequence [52]. In humans, it has been demonstrated that miR146b is downregulated in both bone marrow stem cell (BMSC) chondrogenesis [53] and osteogenesis [54]. In addition, it has been demonstrated that miR146b promotes adipogenesis too [55]. Our finding therefore suggests an epigenetic effect of MSM. In addition, our study reports for the first time the involvement of miR146b in the differentiation of mesenchymal stem cells in zebrafish.

Ultimately, our results demonstrate that MSM modulates the expression of genes involved in chondrogenic and osteogenic differentiation, likely by acting on pathways involved in oxidation processes. Our findings also stimulate interesting ideas for future explorations such as evaluating MSM effects on adipogenesis, a process highly related to osteogenesis.

## Conclusions

These findings provide new insights into the mechanism of action of MSM supplementation on bone and cartilage differentiation and strengthen the rationale for MSM supplementation in patients with osteoarticular disorders as a complementary therapeutic intervention targeting mesenchymal stem cells differentiation.

## Data Availability

All data generated or analyzed during this study are included in this published article.

## Abbreviations

MSM: Methylsulfonylmethane
MSCs: Mesenchymal stem cells
iNOS: Nitric oxide synthase
COX-2: Cyclooxygenase-2
IL-1β: Interleukin
SPARC: Osteonectin
SSP1: Osteopontin;
COL1A1: Collagen type 2
COL1A2: Collagen type 2
BGLAP: Osteocalcin
ARS: Alizarin red staining
Nrf2: Nuclear factor erythroid 2-related factor 2
Keap1: Kelch-like ECH-associated protein 1

## References

[1] Firn R. Nature’s chemicals: the natural products that shaped our world. England: Oxford University Press on Demand; 2010.

[2] Joung YH, Darvin P, Kang DY, SP N, Byun HJ, Lee C-H, et al. Methylsulfonylmethane inhibits RANKL-induced osteoclastogenesis in BMMs by suppressing NF-κB and STAT3 activities. Plos One. 2016;11(7):e0159891, doi: 10.1371/journal.pone.0159891. 10.1371/journal.pone.0159891 PMC4957779 27447722

[3] Kim YH, Kim DH, Lim H, Baek D-Y, Shin H-K, Kim J-K (2009). The anti-inflammatory effects of methylsulfonylmethane on lipopolysaccharide-induced inflammatory responses in murine macrophages. Biol Pharm Bull..

[4] Kloesch B, Liszt M, Broell J, Steiner G (2011). Dimethyl sulphoxide and dimethyl sulphone are potent inhibitors of IL-6 and IL-8 expression in the human chondrocyte cell line C-28/I2. Life Sci..

[5] Ahn H, Kim J, Lee M-J, Kim YJ, Cho Y-W, Lee G-S (2015). Methylsulfonylmethane inhibits NLRP3 inflammasome activation. Cytokine..

[6] Oshima Y, Amiel D, Theodosakis J (2007). 213 the effect of distilled methylsulfonylmethane (MSM) on human chondrocytes in vitro. Osteoarthritis Cartilage..

[7] Butawan M, Benjamin RL, Bloomer RJ (2017). Methylsulfonylmethane: applications and safety of a novel dietary supplement. Nutrients..

[8] Cheleschi S, Fioravanti A, De Palma A, Corallo C, Franci D, Volpi N, et al. Methylsulfonylmethane and mobilee prevent negative effect of IL-1β in human chondrocyte cultures via NF-κB signaling pathway. Int Immunopharmacol. 2018;65:129-39. 10.1016/j.intimp.2018.10.004 30316071

[9] Cheng N-T, Meng H, Ma L-F, Zhang L, Yu H-M, Wang Z-Z, et al. Role of autophagy in the progression of osteoarthritis: the autophagy inhibitor, 3-methyladenine, aggravates the severity of experimental osteoarthritis. Int J Mol Med. 2017;39(5):1224-32. 10.3892/ijmm.2017.2934 PMC5403511 28339018

[10] Bi W, Deng JM, Zhang Z, Behringer RR, De Crombrugghe BJNg. Sox9 is required for cartilage formation. Nat Genet. 1999;22(1):85-9. 10.1038/8792 10319868

[11] Goldring MB, Marcu KBJAr, therapy. Cartilage homeostasis in health and rheumatic diseases. Arthritis Res Ther. 2009;11(3):1-16. 10.1186/ar2592 PMC2714092 19519926

[12] Hunter DJ, Bierma-zeinstra S (2019). Seminar osteoarthritis.

[13] Joung YH, Lim EJ, Darvin P, Chung SC, Jang JW, Do Park K, et al. MSM enhances GH signaling via the Jak2/STAT5b pathway in osteoblast-like cells and osteoblast differentiation through the activation of STAT5b in MSCs. PLoS One. 2012;7(10):e47477. 10.1371/journal.pone.0047477 PMC3469535 23071812

[14] Dalle Carbonare L, Innamorati G, Valenti MTJSCR, Reports. Transcription factor Runx2 and its application to bone tissue engineering. Stem Cell Rev Rep. 2012;8(3):891-7. 10.1007/s12015-011-9337-4 22139789

[15] Kim DN, Joung YH, Darvin P, Kang DY, Sp N, Byun HJ, et al. Methylsulfonylmethane enhances BMP-2-induced osteoblast differentiation in mesenchymal stem cells. Mol Med Rep. 2016;14(1):460-6. 10.3892/mmr.2016.5274 27175741

[16] Aljohani H, Senbanjo LT, Chellaiah MAJPo. Methylsulfonylmethane increases osteogenesis and regulates the mineralization of the matrix by transglutaminase 2 in SHED cells. PLoS One. 2019;14(12):e0225598. 10.1371/journal.pone.0225598 PMC6894810 31805069

[17] Cheng A, Genever PGJJoB, Research M. SOX9 determines RUNX2 transactivity by directing intracellular degradation. J Bone Miner Res. 2010;25(12):2680-9. 10.1002/jbmr.174 20593410

[18] Li X, Huang M, Zheng H, Wang Y, Ren F, Shang Y, et al. CHIP promotes Runx2 degradation and negatively regulates osteoblast differentiation. J Cell Biol. 2008;181(6):959-72. 10.1083/jcb.200711044 PMC2426947 18541707

[19] Shen R, Chen M, Wang Y-J, Kaneki H, Xing L, O'Keefe RJ, et al. Smad6 interacts with Runx2 and mediates Smad ubiquitin regulatory factor 1-induced Runx2 degradation. J Biol Chem. 2006;281(6):3569-76. 10.1074/jbc.M506761200 PMC2647593 16299379

[20] Liu S, Cheng H, Kwan W, Lubieniecka JM, Nielsen TOJMct. Histone deacetylase inhibitors induce growth arrest, apoptosis, and differentiation in clear cell sarcoma models. Mol Cancer Ther. 2008;7(6):1751-1761. 10.1158/1535-7163.MCT-07-0560 18566246

[21] Wright E, Hargrave MR, Christiansen J, Cooper L, Kun J, Evans T, et al. The Sry-related gene Sox9 is expressed during chondrogenesis in mouse embryos. Nat Genet. 1995;9(1):15-20. 10.1038/ng0195-15 7704017

[22] Ducy P, Zhang R, Geoffroy V, Ridall AL, Karsenty GJc. Osf2/Cbfa1: a transcriptional activator of osteoblast differentiation. Cell. 1997;89(5):747-54. 10.1016/s0092-8674(00)80257-3 9182762

[23] Smith N, Dong Y, Lian JB, Pratap J, Kingsley PD, Van Wijnen AJ, et al. Overlapping expression of Runx1 (Cbfa2) and Runx2 (Cbfa1) transcription factors supports cooperative induction of skeletal development. J Cell Physiol. 2005;203(1):133-43. 10.1002/jcp.20210 15389629

[24] Kim DN, Joung YH, Darvin P, Kang DY, Sp N, Byun HJ (2016). Methylsulfonylmethane enhances BMP-2-induced osteoblast differentiation in mesenchymal stem cells. Mol Med Rep..

[25] Joung YH, Lim EJ, Darvin P, Chung SC, Jang JW, Do Park K, Lee HK, Kim HS, Park T, Yang YM (2012). MSM enhances GH signaling via the Jak2/STAT5b pathway in osteoblast-like cells and osteoblast differentiation through the activation of STAT5b in MSCs. Plos one..

[26] Deiana M, Malerba G, Dalle Carbonare L, Cheri S, Patuzzo C, Tsenov G (2019). Physical activity prevents cartilage degradation: a metabolomics study pinpoints the involvement of vitamin B6. Cells..

[27] Whitlock KE, Westerfield M (2000). The olfactory placodes of the zebrafish form by convergence of cellular fields at the edge of the neural plate. Development..

[28] Kimmel CB, Ballard WW, Kimmel SR, Ullmann B, Schilling TF (1995). Stages of embryonic development of the zebrafish. Developmental dynamics..

[29] Du SJ, Frenkel V, Kindschi G, Zohar Y (2001). Visualizing normal and defective bone development in zebrafish embryos using the fluorescent chromophore calcein. Dev Biol..

[30] Dalle Carbonare L, Mottes M, Cheri S, Deiana M, Zamboni F, Gabbiani D, Schena F, Salvagno GL, Lippi G, Valenti MT (2019). Increased gene expression of RUNX2 and SOX9 in mesenchymal circulating progenitors is associated with autophagy during physical activity. Oxidative Med Cell Longevity..

[31] Park E, Gong E-Y, Romanelli MG, Lee K (2012). Suppression of estrogen receptor-alpha transactivation by thyroid transcription factor-2 in breast cancer cells. Biochem Biophys Res Commun..

[32] Sakata-Haga H, Uchishiba M, Shimada H, Tsukada T, Mitani M, Arikawa T (2018). A rapid and nondestructive protocol for whole-mount bone staining of small fish and Xenopus. Sci Rep..

[33] Ha S-H, Choung P-H. MSM promotes human periodontal ligament stem cells differentiation to osteoblast and bone regeneration. Biochem Biophys Res Commun. 2020;528(1):160–7. 10.1016/j.bbrc.2020.05.097 32466845

[34] Aljohani H, Senbanjo LT, Chellaiah MA (2019). Methylsulfonylmethane increases osteogenesis and regulates the mineralization of the matrix by transglutaminase 2 in SHED cells. Plos one..

[35] Cheleschi S, Fioravanti A, De Palma A, Corallo C, Franci D, Volpi N (2018). Methylsulfonylmethane and mobilee prevent negative effect of IL-1β in human chondrocyte cultures via NF-κB signaling pathway. Int Immunopharmacol..

[36] Ezaki J, Hashimoto M, Hosokawa Y, Ishimi Y (2013). Assessment of safety and efficacy of methylsulfonylmethane on bone and knee joints in osteoarthritis animal model. J Bone Miner Metab..

[37] Ucuncu Y, Celik N, Ozturk C, Turkoglu M, Cetin N, Kockara N, Sener E, Dundar C, Arslan A, Dogan H, Kurt N, Suleyman H (2015). Chondroprotective effects of a new glucosamine combination in rats: gene expression, biochemical and histopathological evaluation. Life Sci..

[38] Kim D, Lee D, Oh D, Jeong HC, Lee S-J, Sohn J, Kim OK, Lee J (2020). A mixture containing fermented achyranthes japonica nakai ameliorates osteoarthritis in knee joints of monoiodoacetate-injected rats. J Med Food..

[39] Robert AW, Marcon BH, Dallagiovanna B, Shigunov P. Adipogenesis, osteogenesis, and chondrogenesis of human mesenchymal stem/stromal cells: a comparative transcriptome approach. Front Cell Dev Biol. 2020;8. 10.3389/fcell.2020.00561. 10.3389/fcell.2020.00561 PMC7362937 32733882

[40] Hedbom E, Antonsson P, Hjerpe A, Aeschlimann D, Paulsson M, Rosa-Pimentel E, Sommarin Y, Wendel M, Oldberg A, Heinegård D (1992). Cartilage matrix proteins. An acidic oligomeric protein (COMP) detected only in cartilage. J Biol Chem..

[41] Shen T, Alvarez-Garcia O, Li Y, Olmer M, Lotz MK (2017). Suppression of Sestrins in aging and osteoarthritic cartilage: dysfunction of an important stress defense mechanism. Osteoarthritis Cartilage..

[42] Roughley PJ, Mort JS (2014). The role of aggrecan in normal and osteoarthritic cartilage. J Exp Orthop..

[43] Thatcher EJ, Paydar I, Anderson KK, Patton JG (2008). Regulation of zebrafish fin regeneration by microRNAs. Proc Natl Acad Sci..

[44] Pasqualetti S, Banfi G, Mariotti M (2012). The zebrafish scale as model to study the bone mineralization process. J Mol Histol..

[45] Huang W, Yang S, Shao J, Li Y-PJFibaj, library v. Signaling and transcriptional regulation in osteoblast commitment and differentiation. Front Biosci. 2007;12:3068. 10.2741/2296 PMC3571113 17485283

[46] Flores MV, Tsang VWK, Hu W, Kalev-Zylinska M, Postlethwait J, Crosier P, Crosier K, Fisher S (2004). Duplicate zebrafish runx2 orthologues are expressed in developing skeletal elements. Gene Exp Patterns..

[47] Li N, Felber K, Elks P, Croucher P, Roehl HHJDd. Tracking gene expression during zebrafish osteoblast differentiation. Dev Dyn. 2009;238(2):459-466. 10.1002/dvdy.21838 19161246

[48] Yang D-C, Tsai C-C, Liao Y-F, Fu H-C, Tsay H-J, Huang T-F, et al. Twist controls skeletal development and dorsoventral patterning by regulating runx2 in zebrafish. PLoS One. 2011;6(11):e27324. 10.1371/journal.pone.0027324 PMC3210159 22087291

[49] Boutros T, Chevet E, Metrakos PJPr. Mitogen-activated protein (MAP) kinase/MAP kinase phosphatase regulation: roles in cell growth, death, and cancer. Pharmacol Rev. 2008;60(3):261-310. 10.1124/pr.107.00106 18922965

[50] Son Y, Cheong Y-K, Kim N-H, Chung H-T, Kang DG, Pae H-OJJost. Mitogen-activated protein kinases and reactive oxygen species: how can ROS activate MAPK pathways? J Signal Transduct. 2011;2011. 10.1155/2011/792639 PMC3100083 21637379

[51] Ahn H, Kim J, Lee M-J, Kim YJ, Cho Y-W, Lee G-SJC. Methylsulfonylmethane inhibits NLRP3 inflammasome activation. Cytokine. 2015;71(2):223-31. 10.1016/j.cyto.2014.11.001 25461402

[52] Ordas A, Kanwal Z, Lindenberg V, Rougeot J, Mink M, Spaink HP, et al. MicroRNA-146 function in the innate immune transcriptome response of zebrafish embryos to Salmonella typhimurium infection. BMC Genomics. 2013;14(1):1-15. 10.1186/1471-2164-14-696 PMC3852110 24112639

[53] Budd E, De Andrés MC, Sanchez-Elsner T, Oreffo ROJSr. MiR-146b is down-regulated during the chondrogenic differentiation of human bone marrow derived skeletal stem cells and up-regulated in osteoarthritis. Sci Rep. 2017;7(1):1-11. 10.1038/srep46704 PMC5402270 28436462

[54] Gaus S, Li H, Li S, Wang Q, Kottek T, Hahnel S, et al. Shared genetic and epigenetic mechanisms between the osteogenic differentiation of dental pulp stem cells and bone marrow stem cells. Biomed Res Int. 2021;2021:6697810. 10.1155/2021/6697810 PMC7884974 33628811

[55] Ahn J, Lee H, Jung CH, Jeon TI, Ha TYJEmm. Micro RNA-146b promotes adipogenesis by suppressing the SIRT 1-FOXO 1 cascade. EMBO Mol Med. 528(1):160. 2013;5(10):1602-12. 10.1002/emmm.201302647 PMC3799582 24009212

